# Basophils Orchestrating Eosinophils’ Chemotaxis and Function in Allergic Inflammation

**DOI:** 10.3390/cells10040895

**Published:** 2021-04-14

**Authors:** Joseena Iype, Michaela Fux

**Affiliations:** 1 Clinical Cytomics Facility, University Institute of Clinical Chemistry, University Hospital Bern, Inselspital, CH-3010 Bern, Switzerland; joseenamariam.iype@insel.ch; 2 Institute of Social and Preventive Medicine, University Bern, CH-3012 Bern, Switzerland

**Keywords:** basophils, eosinophils, chemotaxis, eosinophil-basophil interaction, allergic inflammation, eosinophil infiltration, atopic dermatitis, allergic asthma

## Abstract

Eosinophils are well known to contribute significantly to Th2 immunity, such as allergic inflammations. Although basophils have often not been considered in the pathogenicity of allergic dermatitis and asthma, their role in Th2 immunity has become apparent in recent years. Eosinophils and basophils are present at sites of allergic inflammations. It is therefore reasonable to speculate that these two types of granulocytes interact in vivo. In various experimental allergy models, basophils and eosinophils appear to be closely linked by directly or indirectly influencing each other since they are responsive to similar cytokines and chemokines. Indeed, basophils are shown to be the gatekeepers that are capable of regulating eosinophil entry into inflammatory tissue sites through activation-induced interactions with endothelium. However, the direct evidence that eosinophils and basophils interact is still rarely described. Nevertheless, new findings on the regulation and function of eosinophils and basophils biology reported in the last 25 years have shed some light on their potential interaction. This review will focus on the current knowledge that basophils may regulate the biology of eosinophil in atopic dermatitis and allergic asthma.

## 1. Eosinophils and Basophils Are Pivotal Players in the Progression of Allergic Inflammation

Type 2 immunity in the lung and skin is complex involving different types of cells, cells of adaptive and innate immunity, and a multi-layered network of cytokines, chemokines, and effector mediators [1]. Several studies demonstrate that basophils and eosinophils are essential contributors in the initiation, progression, and maintenance of allergic inflammations.

Basophils are the least abundant circulating granulocytes representing 0.5–1% of all leukocytes. They resemble mast cells in several characteristic features, especially in the expression of the high-affinity immunoglobulin E (IgE) receptor FcεRI on their cell surface, which directly binds to the IgE. The cross-linking of FcεRI and IgE complexes upon allergen challenge leads to the release of immunoregulatory and effector mediators, including Th2 cytokines like interleukin-4 (IL-4) and IL-13, histamine, and leukotriene (LTC_4_) [2]. Due to their resemblance to mast cells and scarcity, basophils have often been considered insignificant and analyzed only as a surrogate of the tissue-dwelling mast cells. However, recent studies revealed the unique functions of basophils in chronic allergic diseases such as allergic rhinitis and asthma [3]. Infiltration of basophils and not mast cells during late-phase allergic reaction in the lungs is pivotal for the severity of the inflammatory response as evidenced by the observation that most histamine positive cells are basophils and the simultaneous absence of mediators like tryptase exclusively secreted by mast cells [4]. Histamine and pro-inflammatory lipid mediators (like LTC_4_) released by activated basophils increase vascular permeability and mucus production and promote contraction of smooth muscle cells surrounding the bronchi thereby promoting bronchoconstriction [2]. A non-redundant function of basophils in the context of allergic inflammation is the rapid secretion of IL-4 and IL-13 at levels greater than any other cells capable of secreting these cytokines, including Th2 cells, mast cells, and eosinophils [5]. The release of IL-13 stimulates goblet cells to produce more mucus and to proliferate [6]. In synergy with IL-13, IL-4 provides additional signals for isotype switching to IgE in B cells [5]. Moreover, it has been shown that IL-4 and IL-13 trigger CCL11 release and vascular cell adhesion molecule-1 (VCAM-1) expression from endothelial cells resulting in infiltration of eosinophils [2]. By producing IL-4 and IL-13, basophils regulate infiltration of eosinophils [7], which is confirmed in a recent study using basophil-deficient mice [8]. Furthermore, basophils also secrete various chemoattractants, including IL-8 (own observation [9]) and CCL5 (RANTES) [7], which promote the influx of potent inflammatory cells, neutrophils, eosinophils, and macrophages. Several lines of evidence from murine models suggest basophils’ direct role in antigen presentation for Th2 responses through MHC-II expression [10,11,12], initiation, and maintenance of IgE-mediated Th2 responses possibly independent of T cells and mast cells (reviewed in [13]). Despite this evidence from mouse models of the multifaceted roles of basophils in Th2 allergic inflammation, the crucial function of basophils in human allergic diseases remains undefined because of inadequate supporting evidence.

In addition to the infiltration of basophils, migration of eosinophils to inflammation sites is another essential characteristic of allergic inflammation. Eosinophils are also relatively rare (about 1–5% of all leukocytes) circulating granulocytes. The content of eosinophil granules differs from that of basophils. It mainly comprises cationic proteins such as major basic protein (MPB), eosinophil cationic protein (ECP), eosinophil peroxidase, and eosinophil-derived neurotoxin [2]. However, some of the compounds secreted by eosinophils are also found in basophils, such as IL-4 and IL-13 [2]. In the context of allergic inflammation, the release of cationic mediators has been shown to cause injury to epithelial tissue damage and thereby contribute to airway hyperresponsiveness [14]. MBP released by eosinophils may cause a positive feedback loop since it provokes histamine release and LTC_4_ from basophils [15].

Basophils and eosinophils are present at sites of allergic inflammation in the airways and skin [4,16,17,18,19,20,21]. Namely, increased numbers of basophils are reported in the lung tissues of fatal asthmatics [16], in sputum and bronchoalveolar lavage (BAL) fluid from asthmatics after allergen challenge [4,20]. Interestingly, upon allergen challenge, the increase in basophil percentages in BAL fluids negatively correlate with basophil percentages in the blood, indicating the recruitment of basophils from the circulation into the lung [20]. The presence of basophil infiltrates in atopic allergic inflammation in human skin was established using basophil granule-specific monoclonal antibody (mAb) [18]. In an extension of this observation, Ying and co-workers demonstrated, besides basophils, eosinophils infiltrate 6 h after allergen challenge in late-phase reaction [22]. Consistently, concomitant recruitment of basophils and eosinophils is reported in the skin lesions of eosinophilic pustular folliculitis and atopic dermatitis [21,23]. In asthma, elevated levels of eosinophil count and their mediator, MBP in BAL fluid from asthmatics are also reported [24]. A comparative study between basophils and eosinophils in atopic asthmatics identifies their presence in bronchial biopsies, and their numbers are further enhanced after allergen challenge in the late-phase of asthmatic and skin reactions [17]. Similarly, an increase in numbers of both airway basophils and eosinophils in atopic asthmatics’ sputum after allergen inhalation challenge is observed [19]. Interestingly, it has been demonstrated that eosinophil-derived MBP directly activates the release of histamine and LTC_4_ from human basophils [15,25]. Consistent with this observation, in several experimental allergy models, basophils and eosinophils appear to be closely linked by directly influencing each other or indirectly due to being responsive to similar cytokines and chemokines [7,8,26]. Therefore, it is reasonable that basophils and eosinophils interact with each other at sites of allergic inflammation. In 1995, Thomas et al. reviewed the potential functional network between basophils and eosinophils [27]. New findings in recent years give some more insights into the mechanisms of interaction between these two types of granulocytes. Eosinophils are increased in allergic inflammation and are often considered the main actors of the late phase of allergic rashes. However, although present at low numbers, basophils seem to be pivotal for allergic inflammation progression and maintenance. We will summarize whether and how basophils can regulate the function of eosinophils in allergic dermatitis and asthma.

## 2. Chemotaxis

There are several potential mechanisms on how cells of different types may regulate each other. One of them is undoubtedly the attraction of a particular cell type from circulation to a specific tissue guided by an increasing gradient of chemoattractants. This type of cell migration is known as chemotaxis. It is well established that tissue-residing mast cells play an essential role in triggering infiltration of eosinophils and basophils to sites of inflammation (reviewed in [28]). Moreover, IL-5, produced by allergen-specific T-cells, is considered a key molecule of eosinophil chemotaxis [29,30], whereby systemically delivered IL-5 seems to be superior to IL-5 expressed locally in the airways on impacting airway eosinophilia [31]. Whether basophils and eosinophils attract each other to sites of inflammation such as irradiated skin lesions or inflamed airways is less well documented. Nevertheless, studies using basophil and eosinophil-depleted mice shed some light on this topic and will be summarized in the next section (Table 1 and Table 2).

### 2.1. Contribution of Basophils to Regulate Eosinophil Infiltration to Inflamed Skin

By subcutaneous injection of TNP-conjugated ovalbumin into anti-TNP IgE transgenic mice [40], Mukai and co-workers were able to induce an immediate as well as a late and chronic phase of IgE-mediated chronic allergic dermatitis (IgE-CAI) as evident by infiltration of granulocytes and ear swelling 2–4 days after injection. Interestingly, the induction and progression of early, late, and chronic allergic inflammation was not dependent on T cells, NK-T cells, and NK cells. In contrast, mast cells impacted granulocyte infiltration during the immediate and late but not the chronic phase of allergic skin inflammation [32]. Basophil-depleted mouse models did not exist at that time point. However, some indirect evidence that basophils are regulators of eosinophil infiltration during the chronic phase of allergic inflammation was given by the fact that transfer of the DX5 (CD49b) positive cell fraction, consisting 15–25% of basophils, into FcγR-/- mice restored IgE-CAI [32].

More direct evidence that basophils regulate eosinophil infiltration and disease progression in IgE-CAI is given in a murine model in which basophils were depleted by the usage of the basophil specific anti-CD200R3 antibody (Ba103). Depletion of basophils inhibited the infiltration of eosinophils and the development and maintenance of IgE-CAI. In contrast, in passive cutaneous anaphylaxis, mediated mainly by mast cells, and contact hypersensitivity, a model for type IV hypersensitivity, the clinical symptoms and eosinophil infiltration were not affected by basophil depletion [33]. These findings are in line with studies performed in constitutively basophil-depleted *Mcpt8Cre* BAC transgenic mice [34].

Expressing the human diphtheria toxin (DT) receptor under *CD203* or the eosinophil peroxidase promotor, basophil, and eosinophil depletion, respectively, was obtained upon administration of DT [35]. IgE-CIA was abolished in basophil-depleted BasoDTR mice, confirming the essential role of basophils to control eosinophil infiltration. Besides, Th2 cytokines (IL-4, IL-13, IL-6) and Th1 cytokines (IFNγ and IL-12) were reduced. Notably, CCL24 (eotaxin-2) expression, known to regulate eosinophils’ infiltration, was significantly decreased in BasoDTR mice. In eosinophil-ablated EoDTR mice, IgE-CIA was diminished as well. Unfortunately, whether the attraction of basophils to inflamed skin was affected in the EoDTR mice was not addressed. Nevertheless, although the cytokines as mentioned above were expressed to a lesser degree in EoDTR mice compared to wild-type mice, EoDTR mice showed higher amounts of these cytokines than BasoDTR mice suggesting that basophils initiate and eosinophils ameliorate IgE-CIA [35].

In line with the IgE-CAI mouse models are reported data of croton oil-induced irritant contact dermatitis (ICD). Basophils infiltrated lesion skin within 3 h, followed by eosinophil migration at 24 h upon induction of ICD, suggesting that basophils have the capacity to imprint attraction of eosinophils by the simple fact that basophil infiltration occurs earlier than the one of eosinophil. Further analysis in anti-FcεRIα antibody (MAR-1)-treated and basophil-depleted Bas TRECK mice confirmed a significant reduction of eosinophils in basophil-depleted mice [7].

Therefore, these mouse studies point to basophils’ vital role in regulating eosinophil infiltration to irradiated skin lesions during chronic allergic dermatitis. However, in contrast to these mouse models, in atopic asthma patients in whom late-phase skin reaction was induced, eosinophil infiltration to inflamed skin occurred prior to basophil infiltration, whereby maximal numbers of eosinophils were already observed at 6 h followed by basophils infiltration at 24 h [17,22]. In skin lesions of atopic dermatitis, the accumulation of both basophils and eosinophils are reported [21,36]. It has been widely reported that eosinophil recruitment is associated with increased expression of adhesion molecules, including VCAM-1 [41,42] and specific chemotactic factors such as CCL11 [43]. Interestingly, in basophil-depleted mice, eosinophil infiltration was found abrogated, and furthermore, the basophil-specific deletion of IL-4 showed diminished VCAM-1 up-regulation on endothelial cells [8]. Consistently, the accumulation of basophils that express IL-4 in skin lesions of atopic dermatitis patients was observed [36]. Taken together, it is plausible that infiltrated basophil represent a source of IL-4 that aids in eosinophil recruitment in atopic dermatitis. However, further investigations are necessary to better understand the functional significance of skin-infiltrating basophils in human atopic dermatitis.

### 2.2. The Controversial Role of Basophils in Affecting Eosinophil Infiltration to Sites of Inflammation in the Airways

Findings in mouse models of allergic airway inflammation are less consistent than the ones of IgE-CAI and chronic contact dermatitis. For instance, a mouse study of OVA-induced late asthmatic response revealed that basophil infiltration to sites of allergic airway inflammation and the release of IL-4, IL-5, and IL-13 into BAL fluids occurs before eosinophil, T cell, and monocyte infiltration [37]. However, although depletion of basophils and mast cells by anti-FcεRIα MAR-1 antibody reduced the early phase of specific airway resistance (sRAW), the late phase of sRAW and late eosinophil infiltration to lung tissue was not affected [44]. Consistently, using the basophil-depleted *Mcpt8Cre* BAC transgenic mice, Ohnmacht and their research team demonstrated that the infiltration of eosinophils was not abrogated upon OVA challenge [34]. In contrast, MAR-1 and Ba103 treatment resulted in a significant reduction of eosinophil number in BAL fluid and airways, and reduced IL-4 expression in lung tissue in the study conducted by Zhong et al. [38]. Besides, whether basophils have the capacity to contribute to eosinophil infiltration seems to not only depend on the approach of basophil depletion, but also on the allergen used to induce airway inflammation. For instance, Hammad and co-workers compared the effects of the MAR-1 and Ba103 antibody, respectively, on the infiltration of eosinophils and Th2 cytokine secretion in BAL fluids in an allergic airway inflammation models using house dust mite (HDM) [39]. They found that MAR-1 antibody administration, which recognizes FcεRIα expressed on basophils, dendritic cells, and mast cells, resulted in a significant reduction of eosinophil infiltration and Th2 cytokine production. In contrast, treatment with the basophil specific anti-CD200R3 antibody Ba103 did not significantly affect Th2 cytokine release in BAL fluids. Furthermore, it resulted in a less pronounced but still significant reduction of eosinophil infiltration compared to MAR-1 treated mice [39]. Whether eosinophil depletion affects infiltration of basophils has not been studied in detail. However, interestingly enough, in an HDM-asthma model, it has been shown that eosinophil depletion by the usage of anti-CCR3 antibodies resulted in an 80 to 85% reduction of eosinophils in BAL and lung but did not affect numbers of Th2 cells and airway remodeling. These findings were confirmed in ∆dblGATA eosinophil-ablated mice [45].

In general, basophils’ role in regulating eosinophils’ trafficking seems different in allergic skin and airway inflammation. The reasons for this are manifold and certainly include the fact that allergic asthma and atopic dermatitis are two separate diseases with different underlying mechanisms. In addition, conflicting results can be explained by the undeniable fact that mouse models do not always represent human diseases. We refer the reader to the review of Graham et al. summarizing allergic mouse models and their contribution to understand the human diseases [46].

### 2.3. Overview of the General Mechanisms of Eosinophil and Basophil Chemotaxis

**Figure 1:**
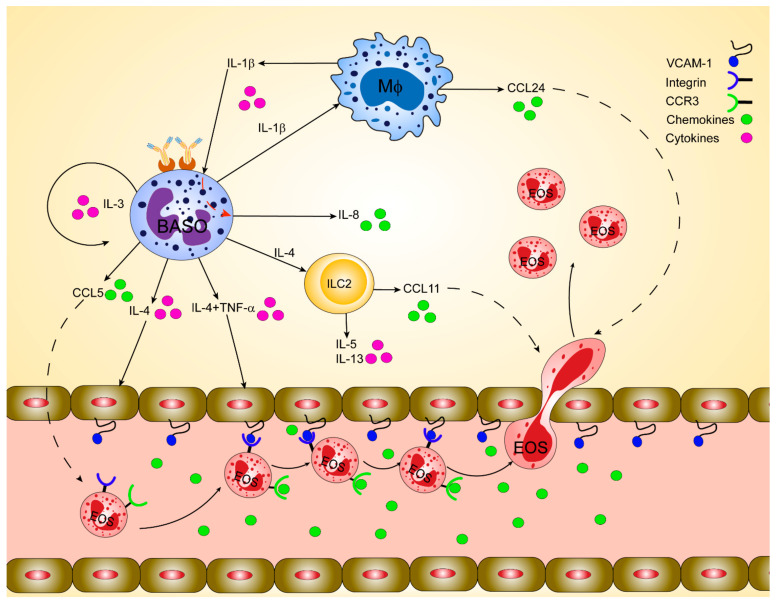
Orchestration of basophil-mediated eosinophil recruitment during allergic inflammation in humans. Basophils play a crucial immunoregulatory role in releasing cytokines such as interleukin (IL)-4 and IL-13 at the inflammatory tissue foci. Basophils-derived IL-4 induces the upregulation of the adhesion molecules like vascular cell adhesion molecule-1 (VCAM-1) on endothelial cells, which facilitates eosinophil infiltration. Basophil-derived IL-4 in synergy with tumor necrosis factor-alpha (TNF-α) stimulates fibroblasts and endothelial cells to produce eotaxins, including CCL11 and CCL24, which can, in turn, attract CCR3positive eosinophils. IL-4 activates lung-resident group 2 innate lymphoid cells (ILC2) to release IL-5, IL-13, and CCL11, stimulating eosinophils’ infiltration. IL-13 stimulates airway macrophages to release CCL24, which regulates eosinophil migration to the airways. Infiltrated inflammatory cells like macrophages and monocytes in the airways activate and secrete IL-1β, which in turn induce basophils to release the potent neutrophil chemoattractant, IL-8. IL-3 secreted by T cells, mast cells, or even basophils themselves induces the expression of CCL5 in basophils, which in turn regulates the trafficking of eosinophils.

The regulation of eosinophils’ chemotaxis has been investigated in many studies. Before we describe the potential mechanism of how basophils may guide the infiltration of eosinophils to inflamed skin, we will shortly outline the most general means of chemotactic regulation of basophils and eosinophils (also summarized in Figure 1).

### 2.4. The CCR3/CCL11/CCL24 Axis

CCL11 (eotaxin-1), CCL24 (eotaxin-2), CCL26 (eotaxin-3), CCL5 (RANTES), CCL3 (MIP-1α), and CCL7, CCL8, CCL13 (MCP-3, -2 and 4), all recognized by CCR3, are prominent eosinophil-active chemokines. CCR3 is constitutively expressed on eosinophils from mice [47] and humans [48] and on basophils from humans [49] but not from mice. Murine models of allergic airway inflammation demonstrated the prominent role of CCR3 in regulating infiltration of eosinophils to inflamed lung tissue. Depending on the immunization protocol that has been used to induce airway inflammation, numbers of eosinophils in BAL fluid and lung tissue were modestly [50] or strongly reduced in CCR3-deficient mice [51,52]. It is less well known whether CCR3 regulates infiltration of basophils to inflamed airways to a similar extent as eosinophils since basophils from mice do not express CCR3. Nevertheless, Sénéchal and co-workers demonstrated in a mouse model of human skin xenografts and autologous transfer of PBMC from allergic donors that basophil infiltration to inflamed skin was not affected by anti-CCR3 antibody administration prior to allergen challenge [53].

CCL11, CCL24, and CCL26 are high-affinity ligands of CCR3. The levels of plasma CCL11 are significantly increased in atopic dermatitis patients [54] and during asthma exacerbation compared to the stable phase of asthma [55]. Moreover, elevated levels of CCL11 in plasma seem to have functional consequences since they are inversely associated with predicted FEV1 [56]. In line with this, is the observation that in the airways of asthma patients compared with healthy controls mRNA of CCL11 and CCL24 are increased at steady state. However, in contrast to CCL26 mRNA, CCL11 and CCL24 mRNAs are not further upregulated in the airways upon allergen challenge [57]. The same accounts for atopic dermatitis patients in whom serum levels of CCL26 correlate with disease severity while CCL24 does not [58].

Interestingly, CCL11 and CCL24 might have different importance in health and disease. On the one hand, CCL11, which, in humans, is constitutively expressed in the small intestine and colon and to a lesser extent in heart, kidney, and pancreas [59,60,61], seems to be more prominent than CCL24 in regulating eosinophil infiltration to the gastrointestinal tract at baseline in healthy state [62,63]. CCL24, on the other hand, seems to dominate the regulation of eosinophil infiltration under pathophysiological conditions. For instance, CCL24 and CCL11/CCL24 double-deficient mice show fewer eosinophils in BAL fluids 20 h upon OVA-induced experimental airway inflammation, while numbers of eosinophils in CCL11 knockouts were comparable to wild-type control mice [40]. This might be explained by the fact that in contrast to CCL11, the expression of CCL24 is prolonged up to 120 h upon allergen challenge and thus has the capacity to regulate eosinophil infiltration at later time points. Consistently, targeted gene disruption of CCL11 results in reduced numbers of eosinophils in BAL fluid after 18 h but not after 48 h OVA-challenge [64]. In terms of basophil chemotaxis, in vitro tests demonstrated that CCL11 and CCL24 are chemoattractants of human basophils, whereby CCL11 seems to be more potent than CCL24 in attracting basophils [49,65,66]. Eosinophil- and basophil-active chemotaxis of CCL11 and CCL24 and their temporal expression was confirmed in humans upon cutaneous allergen challenge. However, expression of CCR3, CCL11, and CCL24 did not correlate with numbers of basophils upon cutaneous allergen challenge questioning whether the eotaxin-CCR3 axis is essential in regulating infiltration of basophils to inflamed skin in humans [22].

Besides the eotaxin-family, the other known agonists of CCR3 have been demonstrated to impact eosinophil and basophil attraction. For instance, increased concentrations of CCL5 and CCL3 have been detected in BAL fluid of asthma patients compared to healthy non-smokers [67]. The chemotactic capacity of these ligands has been demonstrated by in vitro tests using trans-wells in whichBAL fluid was added to one side and purified eosinophils from asthma patients to the other side. Importantly, the usage of anti-CCL5 and anti-CCL7 antibody demonstrated inhibition of eosinophil chemotaxis [67].

There are several recent reviews summarizing findings on the regulation of eosinophil and basophil chemotaxis to inflamed skin and lung and the further effects of the corresponding chemokines such as induction of granulocyte degranulation [68,69].

### 2.5. The GM-CSF Cytokine Family Axis

Members of the GM-CSF cytokine family (IL-3, IL-5, and GM-CSF) tremendously affect the maturation, development, and biology of eosinophils and basophils. Several studies investigated the contribution of IL-3, IL-5, and GM-CSF on chemotaxis (reviewed [70]). For instance, IL-5 and GM-CSF are found elevated in the BAL fluids from allergic subjects after the antigen challenge [71,72,73]. Interestingly, it has also been observed that eosinophils from BAL fluid of antigen challenged-allergic subjects showed enhanced transendothelial migration, suggesting eosinophils are activated in vivo by IL-5 and GM-CSF [74].

Receptors for all three GM-CSF cytokine family consist of a cytokine specific α-chain (IL-3Rα, IL-5Rα, or GM-CSFRα) and a common β-chain [75]. Both blood basophils and eosinophils express receptors for all members of the GM-CSF cytokine family. The sensitivity and magnitude of cell’s responses elicited by different GM-CSF family members are different and dependent on the density of surface expression of the corresponding receptor α-chains on basophils and eosinophils [76]. For instance, IL-3Rα mRNA is abundantly expressed in basophils, whereas in eosinophils, IL-5Rα mRNA is dominant among all the α-chains of three GM-CSF cytokine receptors [77]. Consistently, among the three GM-CSF cytokines, IL-3 potently activates various basophil functions, whereas IL-5 efficiently activates eosinophils [76].

IL-3, IL-5, and GM-CSF play an important role in adhesion and migration of basophils and eosinophils across endothelial cells via the upregulation of β2 integrins (CD11/CD18) expression [74,77,78,79,80,81]. IL-3 was observed to be more potent in upregulating CD11b expression in basophils than in eosinophils, while IL-5 and GM-CSF were almost equipotent in both cell types [79]. In studies using IL-3, IL-5, and GM-CSF cytokines at low concentrations (150 pg/mL), IL-3 seems to be less efficient than IL-5 or GM-CSF on eosinophils’ migration across endothelial cells [74]. On the other hand, at higher concentrations of all the three GM-CSF cytokine members (5 ng/mL), eosinophil migration was equally enhanced [80]. Interestingly, by inhibiting the re-consumption of IL-3 by basophils using a neutralizing antibody, Schroeder et al. demonstrated that basophils release IL-3 upon cross-linking the high-affinity IgE receptor [82]. Hence one may speculate that basophils that infiltrated inflamed tissue attract eosinophils by releasing IL-3 upon allergen binding.

IL-3, IL-5, and GM-CSF effectively prime eosinophils for enhanced chemotactic responses to various stimuli such as platelet-activating factor, leukotriene B4, and complement C5a [83]. The most characterized synergism to promote eosinophilia is the interaction between IL-5 and eotaxin. Studies conducted on eosinophil trafficking in animal models indicate that IL-5 and eotaxin synergistically regulate eosinophils’ migration during allergy [84,85,86].

### 2.6. Mechanisms of How Basophil Imprint Eosinophil Migration to Inflamed Skin and Lung

Basophils constitute only a minor population infiltrating the tissue compared to eosinophils. For instance, in IgE-CAI basophil numbers, which were identified as FcεRIpos, c-Kitneg, accounted for only 2% of infiltrating cells during the chronic phase, in contrast to eosinophils that accounted for the majority of infiltrating cells, namely 40% [32]. Therefore, it is justified to ask how such a minority can have such a significant influence. The study of Cheng et al. gives some more mechanistic insights into basophil and eosinophil infiltration dependency. In three different-IgE-dependent models of atopic dermatitis, basophil-derived IL-4 was shown to induce VCAM-1 expression on endothelial cells of the skin, which resulted in infiltration of eosinophils [8]. Consistently, by using transgenic basophil-depleted mice, eosinophil infiltration was abrogated. Moreover, by eliminating IL-4 from basophils, the study demonstrates the non-redundant role of IL-4-producing basophils in upregulating VCAM-1 on endothelial cells and regulating infiltration of eosinophils. These findings are consistent with the fact that the interaction between α4β1 integrin, expressed on eosinophils and VCAM-1, displayed by endothelial cells, plays a significant role in the adhesion of eosinophils to tissue (reviewed in [87]). Thus, basophil-derived IL-4 might be a possible mechanism for how eosinophil infiltration to inflamed skin is regulated. Moreover, intranasal injection of soluble IL-4R before OVA-challenge reduced VCAM-1 expression on endothelial cells of blood vessels in the airways and blocked eosinophil infiltration [88]. Consistently, in IL-4 deficient mice, fewer eosinophils were observed in BAL fluid than in wild-type mice [89]. Besides, it is well established that in synergy with TNF-α, IL-4 promotes the release of CCL11 [90,91], CCL24 [92], and CCL26 [93] from endothelial cells and fibroblasts. Unfortunately, basophils have not been addressed in these studies. Notably, however, an increased level of IL-4 concomitantly with basophils infiltration upon parasite infection was observed, and basophils are known to be the major source of IL-4 [94,95,96,97]. Interestingly, induction of eotaxin-family members is STAT6 dependent [98,99]. Voehniger et al. observed in lung tissue a non-T non-B cell population that produce IL-4, which was responsible for eosinophil infiltration and dependent on STAT6 [96]. One may therefore assume that basophil-derived IL-4 induces eotaxin-family members in a STAT6-dependent manner, which in turn results in eosinophil infiltration. A papain-induced airway inflammation model further gives evidence that basophils indirectly trigger eosinophil infiltration. Namely, basophil-derived IL-4 activated lung-resident group 2 innate lymphoid cells (ILC2s) to release IL-5, IL-13, and CCL11, which stimulate infiltration of eosinophils [26].

Besides IL-4, basophils are known to produce IL-13 upon FcεRI cross-linking [100,101]. Pope and their research team demonstrate that intratracheal injection of IL-13 into CCL24-deficient mice resulted in reduced numbers of eosinophils in BAL fluid. Interestingly, macrophages in the airway lumen seemed to be the major source of CCL24 [63]. It is therefore quite reasonable to speculate that basophil-derived IL-13 stimulates airway macrophages to release CCL24, which regulates eosinophil recruitment to the airways.

Further, in vitro studies revealed that basophils regulate eosinophils’ infiltration in co-operation with fibroblast, whereby basophil-derived TNF-α and IL-4 seem to stimulate CCL11 expression in fibroblast, which in turn attract eosinophils through CCR3. Furthermore, IL-3 induces high expression of CCL5 in basophils, which is further enhanced by co-culturing of basophils with fibroblast and basophil-derived TNF-α [7]. It is, therefore, likely that fibroblast-derived CCL11 and IL-3-mediated induction of CCL5 regulate the trafficking of eosinophils [102]. The importance of IL-3 in regulating attraction of eosinophils to airways is further given by the fact that IL-3 is a very potent inducer of IL-4 and IL-13 from human basophils [76,103,104]. Interestingly, basophil-derived IL-4 and IL-13 can be inhibited by a blocking anti-IL-3Rα antibody [76] and INF-α treatment [104]. Hence, it would be interesting to investigate whether, under such treatments, eosinophil infiltration can be blocked. Moreover, IL-1β induces in synergy with IL-3 the release of IL-8 (own observation [9]), which was shown to cause eosinophil chemotaxis [105] and thus provides another mechanism basophil regulate recruitment of eosinophils.

Besides cytokines and chemokines, basophil specific proteases contribute to the recruitment of eosinophils to inflamed tissues. Namely, mouse mast cell proteases (mMCP)-8, which is expressed explicitly in mouse basophils, triggers leukocyte infiltration by upregulating the expression of CCL24 (reviewed in [106]). Interestingly, mMCP-8 is highly homologous to human Granzyme B, which is released upon activating IL-3-primed basophils with various agonists (anti-FcεRIα, C5a, fMLP) [107]. Whether Granzyme B contributes to eosinophils chemotaxis in humans has, however, not yet been addressed. In addition, mMCP-11, a protease preferentially expressed in basophils, seems to attract eosinophils indirectly by proteolytic cleavage of a yet unidentified serum protein, whose product triggers recruitment of eosinophils. Consistently, mMCP-11-deficient mice show a diminished IgE-CIA. Curiously, however, mMCP-11-deficient basophils produce IL-4 in a normal range upon MCP-2 stimulation (reviewed in [106]), indicating that other mechanisms than IL-4-dependent induction of VCAM-1 contribute to eosinophil infiltration.

## 3. Cell-to-Cell Interaction at Sites of Inflammation

Another obvious mechanism, besides chemotaxis, in which cells of different origins may influence each other is the cell-to-cell contact. A recent book chapter by Landolina et al. summarizes the interaction of mast cells and eosinophils. The authors describe the formation of an allergic effector unit (AEU), enabling an enhanced function of both cell types [108]. In addition, studies have demonstrated that basophils and fibroblasts/endothelial cells directly interact. For instance, direct intercellular contact between basophils and fibroblasts was needed to mediate basophil-derived IL-8, CCL2, and CCL5 secretion upon NOD2/TLR2 stimulation [109]. Moreover, FcεRI and integrin signaling resulted in clustering of basophils at endothelial junctions upon allergen challenge in IgE-DNP sensitized mice. This arrest allowed the local increase of basophil-derived IL-4, which activates IL-4 receptor-expressing endothelial cells to upregulate VCAM-1 [8]. Consistently, the work of Schroeder and colleagues suggests that cell-to-cell interaction between basophil and lung epithelial cells triggers histamine, IL-4, and IL-13 release from basophils [110]. Moreover, studies on whether basophils may act [10,12,111,112] or not [113] as antigen-presenting cells (APC) stimulated the interest in investigating whether and if so, how basophils interact with T cells and dendritic cells via cell-to-cell contact. For instance, by using YEP fluorescence expressing basophils and OVA-specific T cells that expressed the red fluorescence protein DsRed, Sullivan and co-workers demonstrated that basophils interact with T cells in lung tissue but not in lymph nodes upon an *N. brasiliensis* infection [114]. These findings challenged, on one side, the role of basophils as APC in lymph nodes, but underscore that basophils interact with T cells by direct cell-to-cell contact resulting in worsening of Th2 inflammation [115]. Several studies published a few years later further investigated the interaction between basophils and different subpopulations of T cells in variable inflammatory conditions in more detail (reviewed in [116]).

Unfortunately, however, whether basophils and eosinophils have direct cell-to-cell interaction during allergic inflammation is poorly defined. Nevertheless, the simultaneous presence of basophils and eosinophils at sites of inflamed skin was observed in chronic allergic dermatitis (ICD) patients giving evidence of basophil and eosinophil couple formation in vivo. Additionally, the same study confirmed the vicinity of basophils and eosinophils at inflammatory foci in an IgE-CAI mouse model [33]. Consistently, basophils promote activation of eosinophils by direct cell interaction, which induces activation markers (CD69, CD86, and intercellular adhesion molecule-1) on eosinophils [7]. Recently, basophils, eosinophils, and T cells have been found to co-localize in lung tissue of COPD patients [117]. These few studies imply that eosinophils and basophils co-localize in inflamed tissue and may affect each other’s function by cell-to-cell contact. However, currently, the volume of research on that topic is small, not allowing sound conclusions. Moreover, direct evidence that basophil and eosinophils form a network of soluble mediators and the corresponding receptors are mainly missing, too. It is reasonable to speculate that a soluble mediator–receptor network exists between these two types of granulocytes simply because both eosinophils and basophils produce and release a plethora of cytokines, chemokines, and growth factors [13,118], as stated elsewhere [27].

## 4. Common Molecular Targets on Basophils and Eosinophils for Novel Therapies in Allergic Inflammation

The simultaneous presence of both basophils and eosinophils has been identified at the inflammation sites in chronic allergic disorders such as dermatitis, and asthma. Although they are considerably different in their ability to respond to stimuli, they share similar characteristics in terms of surface receptors and the inflammatory mediators they secrete. For instance, both basophils and eosinophils respond to inflammatory stimuli such as IL-3, IL-5, IL-33, and generate inflammatory cytokines such as IL-4 and IL-13 during allergic inflammation. Therefore, targeting both basophils and eosinophils together would be an effective anti-allergic therapeutic strategy. In this section, we summarize the possible common molecular targets for basophils and eosinophils.

IL-5-IL-5Rα axis: Three monoclonal antibodies targeting the IL-5-IL-5Rα axis have been developed for clinical purpose. Among them, mepolizumab and reslizumab are humanized mAb that target and neutralize circulating IL-5. Both of them have been approved for the treatment of adults with severe eosinophilic asthma [119]. Though basophil progenitors are unlikely to depend on IL-5 for development, blood basophil counts measured in routine clinical laboratories suggest they decrease following mepolizumab treatment [120]. However, a recent study by Wright et al. has demonstrated that mepolizumab does not alter the blood basophil count, and therefore, clinical benefit is likely to be independent of basophils [121]. Like mepolizumab, reslizumab treatment profoundly decreased circulating eosinophil counts in patients with asthma [122], but its effects on basophils are not investigated. Benralizumab is directed against the membrane-expressed IL-5Rα-chain and induces antibody-mediated cellular cytotoxicity (ADCC) in eosinophils, depleting eosinophils both in blood and tissues [123]. Expression of the IL-5Rα-chain by basophils makes it a potential target of benralizumab. Laviolette et al. showed that blood basophils counts were profoundly reduced with benralizumab treatment, but in this study, basophils have been studied less extensively, and the clinical relevance is unknown [124].

IL-3-IL-3Rα axis: We have previously shown that blocking of the IL-3Rα-chain with the CSL360, a humanized anti–IL-3Rα mAb, significantly ameliorates the basophil phenotype and function [76]. Yet, there are no clinical trials conducted with this mAb. CSL362 is another humanized anti–IL-3Rα mAb derived from clone 7G3 that targets and neutralizes IL-3. As predicted from in vitro and ex vivo studies, CSL362 produced a marked, dose-dependent and sustained depletion of peripheral blood basophils and plasmacytoid dendritic cells (pDCs) [125]. However, there has been no investigation conducted whether CSL362 affect eosinophil blood counts.

Anti-alarmins: Airway epithelium is in constant contact with various external stimuli, such as infectious agents, environmental allergens, and atmospheric pollutants. In response to such stimuli, airway epithelium releases epithelial-derived cytokines, such as IL-25, IL-33 collectively known as “alarmins”. Among them, IL-33 has been shown to activate both basophils and eosinophil phenotype, making them attractive targets for biologics [126]. Monoclonal antibodies targeting IL-33 or its receptor, ST2, are in clinical development, with several in phase 2 trials [127]. Nevertheless, anti-IL-33 and ST2 treatment reduced airway inflammation in a murine model of asthma and found that both treatments reduced the total cell counts and eosinophil counts in BAL fluid and significantly reduced the Th2 cytokine [128]. Recently, Jeannne Allinne et al. conducted a murine study with IL-33 neutralizing antibody REGN3500, which is currently in clinical development to treat asthma and COPD. Anti-IL-33 neutralizing antibody REGN3500 actively reduced the established HDM-induced lung infiltration by eosinophils, ST2positive CD4+ T-cell numbers, and Th2 cytokine levels in lungs, and blocks the further increase in lung neutrophilia and levels of pro-inflammatory cytokines [129]. However, whether anti-IL-33 or anti-ST2 treatment affects basophils has not been investigated in the above-mentioned studies.

Anti-CCR3: The potential of CCR3 as a therapeutic target was established through the observations that CCR3-null mice and eotaxin-1 and eotaxin-2 double-knockout mice displayed significantly reduced allergen-induced airway eosinophil recruitment [130]. A short-term study was conducted in patients with allergic asthma and control subjects using a CCR3 antagonist, AXP1275. No significant effect on the number of circulating eosinophils and sputum eosinophils and metachromatic cells (mast cells and basophils) was observed [131]. R321 is a novel, biased nanoparticle CCR3 antagonist that inhibits G-protein signaling but not β-arrestin-mediated CCR3 internalization and degradation. Intravenously administered R321 significantly reduces eosinophil recruitment into the blood, lungs, and airways and prevents airway hyperresponsiveness in a mouse eosinophilic asthma model [132]. Nevertheless, the effect of R321 on basophils is still unknown.

CRTH2 and PGD2 axis: Chemoattractant receptor-homologous molecule (CRTH2) is highly expressed on human peripheral blood basophils and eosinophils and has been shown to mediate chemotaxis of eosinophils and basophils. Prostaglandin D2 (PGD2), one of the key mediators released by degranulating eosinophils and basophils upon allergen exposure, is the principal ligand of CRTH2. Royer et al. showed that AZ11665362, a novel CRTH2 antagonist, is more potent than Ramatroban (BAYu3405, the only clinically available CRTH2 antagonist) in inhibiting the migration of both human eosinophils and basophils. Besides, AZ11665362 abrogates the PGD2-induced mobilization of eosinophils from the bone marrow of the guinea pig [133]. However, more extensive clinical studies are necessary to understand the clinical relevance of AZ11665362.

Anti-inflammatory cytokines IL-4 and IL-13: Among the recently developed anti-IL-4 and anti-IL-13 biologic drugs, dupilumab is very promising given its ability to inhibit the biological effects of both IL-4 and IL-13. Dupilumab is a human mAb that is directed against the shared α-subunit of the IL-4 receptors. It blocked the signaling from IL-4 and IL-13 and showed efficacy in patients with moderate-to-severe asthma and airway and peripheral eosinophilia [134]. Dupilumab is the only biologic for asthma treatment that simultaneously reduces the severe exacerbations but on the other hand transiently increased blood eosinophilia [135,136,137]. The increase in the blood eosinophils is consistent with the hypothesis that duplimuab blocks the migration of eosinophils into tissue by inhibiting IL-4 and IL-13-mediated production of eotaxin and VCAM-1 expression. Dupilumab was also approved for treatments in adults with moderate-severe atopic dermatitis [138]. However, the result on eosinophil counts from different clinical studies with dipulimab treatment in atopic dermatitis is controversial. For instance, no changes in eosinophil count was observed for dupulimab treatment up to 16 weeks [139]. In contrast, Yamauchi et al. showed ameliorated blood eosinophilia for up to 32 weeks following dupilumab treatment in patients with atopic dermatitis [140]. However, the effects of Dipulimab on basophils has not been investigated.

Small-molecule inhibitors targeting pro-survival proteins: The therapeutic potential of directly inhibiting pro-survival proteins was unveiled with the development of small-molecule inhibitors targeting B cell lymphoma 2 (BCL-2) homology domain 3 (BH3) mimetics. We have shown that ABT-199, an inhibitor for BCL-2, potently induces apoptosis in human basophils but not in human eosinophils [141,142]. More importantly, our previously published studies demonstrated that ABT-263, a selective inhibitor for both BCL-2 and BCL-2-like 1 (BCL-xL), induced cell death of human eosinophils and basophils [141]. However, pre-clinical data predicted a rapid and concentration dependent thrombocytopenia as a side effect of ABT-263 [143].

## 5. Concluding Remarks

We summarized findings on how basophils regulate the infiltration of eosinophils, and there are a few indications that these two types of granulocytes form cell-to-cell contact in tissue. The most recent findings demonstrating that basophils mediate eosinophil infiltration were achieved by mouse and in vitro models [7,8,109] and were published up to seven years ago. Apparently, there is still a considerable gap of knowledge on this research topic. Furthermore, studies on possible targets to attenuate and treat allergic inflammation rarely consider both eosinophils and basophils. This could be due to the technical difficulties in isolating and phenotyping these minority cells at the inflammatory sites. With the advent of new approaches like imaging mass cytometry-based studies could help gain more insights and direct evidence on basophil–eosinophil interactions at inflammatory sites. Considering that identifying which Th2-endotypes is involved in the pathogenicity of allergy seems to be a determinant of whether a specific treatment is successful or not [110], we think it will be essential to study the interaction of basophils and eosinophils in more details in the future. The simultaneous study of basophils and eosinophils could result in better profiling of Th2-endotypes, allowing individualized treatment.

## Figures and Tables

**Table 1:** Mouse models of allergic skin diseases.

Model	Sensitization	Challenge	Way of Basophil Assessment	Diminished Eosinophil Infiltration	Reference
IgE-CAI	i.v. TNP-specific IgE	s.q. TNP-OVA	CD49b^pos^ cell fraction	Yes	[32]
			Anti-CD200R3 (B103)	Yes	[33]
			*Mcpt8Cre* BAC	Yes	[34]
			BasoDTR mice	Yes	[35]
ICD	i.p NAC	NAC and croton oil on skin	Anti-FcεRIα (MAR-1)	Yes	[36]
			BasTRECK	Yes	[36]

IgE-CAI, IgE-chronic allergic inflammation; OVA, ovalbumin; ICD, irritant contact dermatitis; NAC, N-Acetylcystein.

**Table 2:** Mouse models of allergic airway inflammation.

Model	Sensitization	Challenge	Way of Basophil Assessment	Diminished Eosinophil Infiltration	Reference
OVA-induced asthma	i.p. OVA-alum	i.t. OVA	Anti-FcεRIα (MAR-1)	No	[37]
	i.p. OVA-alum	i.n. OVA	*Mcpt8Cre* BAC	No	[34]
			Anti-FcεRIα (MAR-1)	Yes	[38]
			Anti-CD200R3 (B103)	Yes	[38]
HDM-induced asthma	i.n. HDM	i.n. HDM	Anti-FcεRIα (MAR-1)	Yes	[39]
	i.n. HDM	i.n. HDM	Anti-CD200R3 (B103)	Yes	[39]

OVA, Ovalbumin; HDM, house dust mite.
